# Atomic View of Filament Growth in Electrochemical Memristive Elements

**DOI:** 10.1038/srep13311

**Published:** 2015-08-21

**Authors:** Hangbing Lv, Xiaoxin Xu, Pengxiao Sun, Hongtao Liu, Qing Luo, Qi Liu, Writam Banerjee, Haitao Sun, Shibing Long, Ling Li, Ming Liu

**Affiliations:** 1 Key Laboratory of Microelectronics Devices and Integrated Technology, Institute of Microelectronics, Chinese Academy of Sciences, Beijing, 100029, China; 2 Lab of Nanofabrication and Novel Devices Integration Technology, Institute of Microelectronics, Chinese Academy of Sciences, Beijing, 100029, China

## Abstract

Memristive devices, with a fusion of memory and logic functions, provide good opportunities for configuring new concepts computing. However, progress towards paradigm evolution has been delayed due to the limited understanding of the underlying operating mechanism. The stochastic nature and fast growth of localized conductive filament bring difficulties to capture the detailed information on its growth kinetics. In this work, refined programming scheme with real-time current regulation was proposed to study the detailed information on the filament growth. By such, discrete tunneling and quantized conduction were observed. The filament was found to grow with a unit length, matching with the hopping conduction of Cu ions between interstitial sites of HfO_2_ lattice. The physical nature of the formed filament was characterized by high resolution transmission electron microscopy. Copper rich conical filament with decreasing concentration from center to edge was identified. Based on these results, a clear picture of filament growth from atomic view could be drawn to account for the resistance modulation of oxide electrolyte based electrochemical memristive elements.

The past four decades have witnessed the boom of the semiconductor industry. Von-Neumann computing, which carries out logical operations by transferring data between logic and memory cells, serves as the core component of today’s information systems^1^. As transistors’ dimensions approach the sub-10 nm scale, the improvements in system performance are slowing down^2^. Alternative computing concepts based on new devices and architectures with logic and memory functions are therefore needed^3^. The memristive device, which is a combination of memory and a resistor, can retain its resistance states as a function of the flowed current or the history of the applied voltage^4^,^5^,^6^. This analog behavior of resistance modulation provides new opportunities for developing alternative computing architectures. Memristive elements have already been shown to have exciting memory performance, such as high speed (<5 ns), high endurance (>10^12^) and excellent scalability (atomic size)^7^,^8^,^9^,^10^. Logic operations beyond conventional silicon transistors and the hardware architecture of neuromorphic computing have also been developed recently^11^,^12^.

Cation migration-based resistive switching devices, also called electrochemical metallization cells, belong to a typical class of memristive elements^6^,^13^. In these devices, a solid electrolyte of an ionic conductor or a mixed ionic/electronic conductor is sandwiched between an electrochemically active electrode (Ag or Cu) and an inert metal electrode (Pt or W)^14^. For successful commercialization of new technologies, fully understanding the underlying physics is required to predict and optimize the device performance.^[1]^ However, due to the stochastic nature and fast growth of localized conductive filament (CF), the physical mechanisms governing the resistive switching remain unclear. Several outstanding studies provided direct images of nanoscale CFs by transmission electron microscopy (TEM)^15^,^16^,^17^,^18^, which had significantly improved the understanding of resistive switching. However, the understanding on the filament composition and its growth kinetics are still the subjects on hot debate. Moreover, most of the reported works utilized specially designed specimens with either thick films (more than 20 nm) or planar structures, which were quite different from the real devices used in array or integrated circuit. In the integrated devices, the switching layer is generally required to scale its thickness down to 2–5 nm (the value commonly used in test chips)^19^,^20^,^21^,^22^. The growth kinetics of filament in this ultra-thin material system is more complex because of the limited space domain. In order to promote the commercialization progress of memristive devices more rapidly, there is an urgent demand to elucidate the switching behavior in the thin material system clearly.

In this study, we investigated the switching behavior of a Cu/(4 nm)HfO_2_/Pt memristive device in a one transistor/one resistor (1T1R) structure, which is a basic unit of storage in practical application. In order to capture the detailed information on the filament growth, we developed a refined method to regulate the current flow through the memristive device in real-time. Using this method, we observed discrete tunneling conduction and quantized conduction during filament growth. The relationship between the filament length and tunneling resistance was analyzed by direct tunneling conduction. The increment of filament length was found to have a unit of feature length, matching with the hopping conduction of Cu ions between the interstitial sites of HfO_2_ lattice. High resolution transmission electron microscopy (HRTEM) was used to characterize the nature of the filament. Copper rich conical filament with decreasing concentration from center to edge was identified. Based on these results, a clear picture of filament growth in oxide based electrolyte was suggested from a view of atomic scale.

## Results

### Real-Time Regulation of Current with Varied Gate Voltage

**Figure 1:**
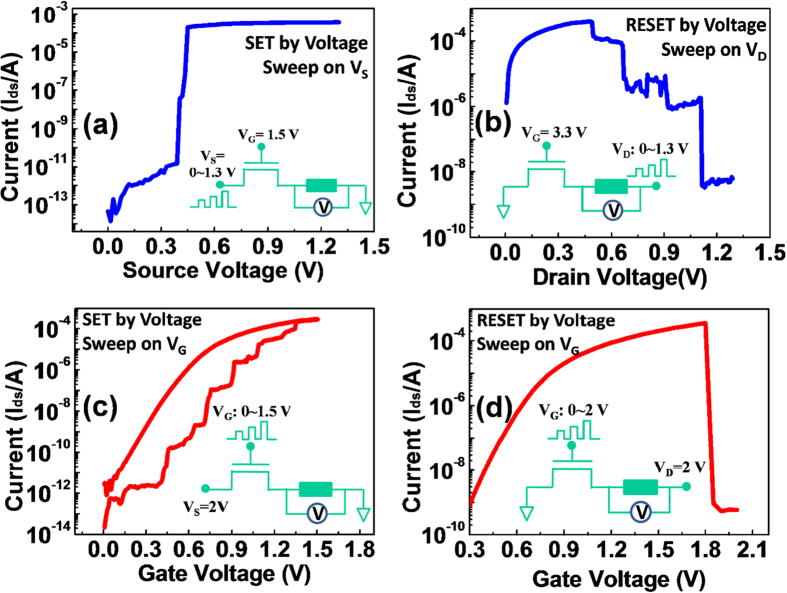
Real-time regulation of the current flow. (**a**) The I-V curve of the common SET operation with V_S_ swept from 0 ~ 1.3 V and V_G_ constantly biased at 1.5 V. (**b**) The I-V curve of the RESET process with V_D_ sweeping. (**c**) The SET curve for the varied V_G_ programing scheme. V_S_ is constantly biased with a voltage of 2 V and V_G_ varies from 0 V to 1.5 V with an increasing rate of 0.005 V per step. (**d**) The I_DS_ dependence of V_G_ in the RESET process, with V_D_ kept at 2 V and V_S_ at ground.

Digging the growth kinetics of filament is always of research interest. The commonly observed fast resistive switching during programming dramatically increases the difficulty in capturing detailed information about filament growth. Figure 1a shows the I-V curve of a typical SET operation (from high resistance state-HRS, to low resistance state-LRS) of a Cu/HfO_2_/Pt device, in which V_S_ was swept from 0 ~ 1.3 V and V_G_ was constantly biased at 1.5 V (detailed information on device fabrication and test conditions could be found in Figure S1). This is a widely used approach to program the electrochemical metallization devices. As V_S_ increased to around 0.5 V, the I_DS_ suddenly jumped from 0.1 nA to 200 μA, which was the saturation current of the transistor under V_G_ = 1.5 V, indicating a sharp decrease in the cell resistance from several GΩ to hundreds of Ω. This fast resistive switching was resulted from positive feedback from the local electrical field^23^,^24^. Once the filament started to grow, the electrical field on the tip was enhanced due to the point discharge effect and the reduction of the distance from the filament tip to the counter electrode. As a result, an abrupt transition was observed. Figure 1b shows the I-V curve of the RESET process (from LRS to HRS) as V_D_ was swept. The I_DS_ decreased with V_D_, showing that the cell resistance was highly dependent on the RESET voltage. The RESET process corresponded to a joule heat assisted dissolution process in the filament, starting from its thinnest point^25^.

To obtain detailed information about the filament growth, we developed a refined method to regulate the programing current in real-time. The gate of the access transistor was applied with a varied V_G_ and the V_S_ was biased with a constant voltage (shown in the insert of Fig. 1c). Initially, the cell showed high resistance of about 100 GΩ, which was lower than the off resistance of the transistor (10^13^ Ω). Almost no voltage dropped on the cell at this time. As V_G_ increased, the resistance of the transistor began to decrease and gradually reached a value comparable to that of the cell. The voltage drop on the cell (V_C_) increased. As long as the V_C_ was high enough for Cu migration, the filament started to grow, corresponding to the cell resistance decreasing.

As shown in Fig. 1c, when the V_G_ was in the vicinity of 0.4 V and the resistance of the cell started to decrease, the transistor actually worked in a sub-threshold region with very limited current (below 0.1 nA). Once the resistance of cell dropped below that of the transistor, the V_C_ reduced simultaneously. The forward growth of the filament depended on the further increase of the gate voltage. As a result, the growth of the CF was greatly retarded in this case. Figure 1d displays the dependence of I_DS_ on V_G_ in the RESET process (with V_D_ kept at 2 V and V_S_ at ground). A sharp resistance change from hundreds of Ω to GΩ was observed, which was resulted from the positive feedback of joule heating^26^.

### Observations of Discrete Tunneling and Quantized Conduction

**Figure 2:**
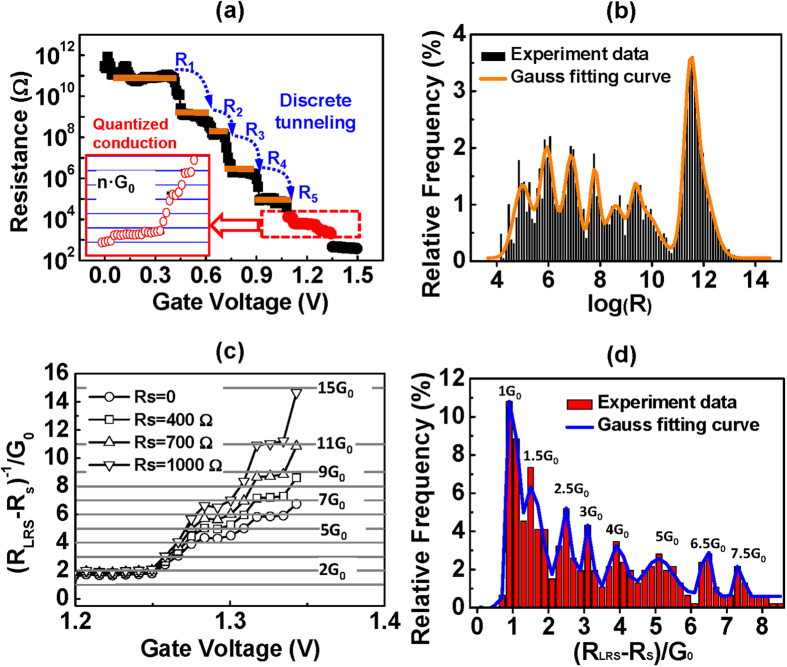
The refined history of the resistance change during programming. (**a**) The resistance of cell as a function of V_G_ in the Fig. 1(c) test. A discrete change in resistance is observed. The insert is the magnification of the red solid dots region. (**b**) Histogram of the resistance states measured from 13360 points in 100 SET curves for V_G_ < 1.1 V. The peaks are fitted with Gaussian distributions as a guide to the eye. (**c**) The fitting results of the red dots region in (**a**) after taking into account the series resistance (R_S_). (**d**) Histogram of the conductance from 20 SET curves of 10 cells after reducing the serial resistance to 700 Ω. The distribution peaks are positioned at integer or half-integer multiples of G_0_, which are fitted with Gaussian distributions as a guide to the eye.

The precise control of the resistance change by real-time regulating the compliance current provided a good platform for studying the growth kinetics of the filament. Figure 2a plots the resistance of cell in the Fig. 1c as a function of V_G_. The voltage of the bottom electrode (V_B_, equal to V_C_) was output to the tester through a detecting point. The cell resistance during programming could then be directly obtained from I_DS_/V_B_. The discrete change in the cell resistance can be clearly observed in Fig. 2a. This trend is more clearly shown in the histogram of the resistance states (Fig. 2b) collected from 13360 measured points in 100 SET curves with V_G_ < 1.1 V. It should be noted that the cell resistance was highly dependent on the V_G_, with a wide range from GΩ to kΩ. Multi-level storage can easily be achieved by controlling different V_G_ (as shown in Figure S2). This large-range resistance distribution cannot be explained by the width variation of the filament or multi-filaments, because the upper limit resistance of atomic-scale filament is only around 12.9 kΩ (1/G_0_). In the tunneling model or the quantum point contact model^27^,^28^,^29^, incomplete filament with a spatial gap spacing from the counter electrode was used to account for the situation of R >>1/G_0_. The gap provides a potential barrier for electron transmission and the resistance exponentially increases with the gap length. A small change of the gap would result in large variation of the tunnel resistance. The temperature dependence measurement is an effective approach to identify the tunneling conduction. Figure S3 shows the measurement of temperature dependence on the cell resistance programmed by V_G_ < 1.1 V. Very week dependence was detected, indicating the tunneling was the dominant conduction of the cell when V_G_ was less than 1.1 V.

As the cell resistance dropped to around 13 kΩ (V_G_ >1.1 V), quantized conduction took place^30^,^31^. The conductance plateaus were positioned at integer or half-integer multiples of the fundamental conductance of G_0_ = 2e^2^/h (where e is the charge of the electron and h is Planck’s constant), as presented by the red dot region of Fig. 2a and its magnification shown in the insert. The series resistance (R_s_) must be taken into consideration to precisely extract the quantized conductance^32^. Here, the R_S_ had a range of 500 to 1000 Ω. The detailed estimation of the R_S_ can be found in Figure S4. The fitting results of the cell conductance for various R_s_ are shown in Fig. 2c. A more concise view of the quantized conductance is shown in the histogram plot of conductance in Fig. 2d, collected from 100 SET curves in 10 cells. The distribution peaks are positioned at integer or half-integer multiples of G_0_, which are fitted with Gaussian distributions as a guide to the eye. This result is in accordance with the observed transition from the tunnel gap region to a quantum contact in a gap-type device, measured by scanning tunneling microscope (STM)^33^. The quantized behavior can be ascribed to quantum effects, when the filament constrictions were of the order of the Fermi wavelength of the electron^30^,^31^,^32^,^34^. The ballistic transport of the carrier through the atomic constriction can be described by the Landauer theory (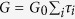, where *τ*_*i*_ is the transmission mode of the *i*_*th*_ eigenmode and the sum is over all occupied modes). As the width of the constriction increased, more conduction modes were allowed. For each additional mode, the conduction of the device jumped by one unit of G_0_. The half-integer quantization may arise from the absorption of defects or electron scattering near the portion of filament that quantized the conductance^31^,^34^.

### HRTEM Characterization on the CF

**Figure 3:**
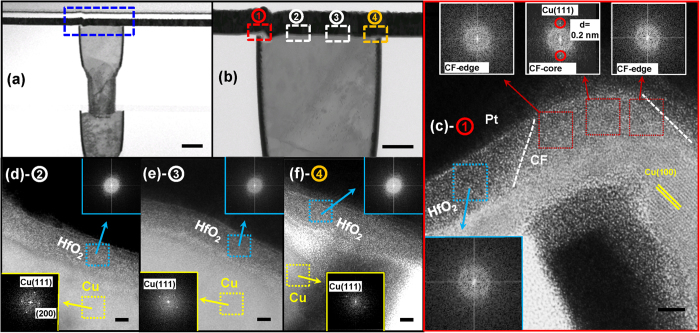
Morphology and structure characterizations of the filament by HRTEM. (**a**) The cross-section of the Cu/HfO_2_/Pt device. (**b**) The magnification of (**a**). (**c**) High resolution image of region ① in (**b**). The upper insertions are the FFT patterns of CF left edge, core edge and right edge. The lower left insertion is the FFT pattern of the HfO_2_ with no CF region. (**d**) A high resolution image of region ② in (**b**). (**e**) A high resolution image of region ③ in (**b**). (**f**) A high resolution image of region ④ in (**b**). The scale bars in (**a**) and (**b**) are 100 nm and in (**c**–**f**) are 2 nm. The yellow and blue framed insertions in (**d**,**e**) are the FFT patterns of the Cu electrode and HfO_2_ layer with no CF, respectively.

In order to clarify the growth kinetics of filament, HRTEM analysis on the physical nature of CF was carried out. After programing the device with a relatively large compliance current (1 mA/V_G_@2.5 V), the device was cut with a focused ion beam and characterized by HRTEM. Figure 3a,b show the cross-section of the Cu/HfO_2_/Pt device and its magnification, respectively. High-resolution images of the cross-section from the left to right corner (regions 1, 2, 3 and 4) are shown in Fig. 3c–f. A conical region with a low contrast in the HfO_2_ layer was found in the left corner. An electron energy-dispersive spectroscopy (EDS) analysis of this area revealed that it contained a large amount of Cu. The feature peak of the Cu signal in this area was much higher than that in other region (as shown in Figure S5), indicating the CF was copper rich. A gradual profile of Cu concentration was observed on the edge of CF, suggesting more copper in the CF center and less copper at the edges. The Cu electrode was found to have a crystalline structure, as indexed by the lattice fringes in Fig. 3c and yellow framed fast Fourier transformation (FFT) patterns in Fig. 3d–f. The HfO_2_ layer was found to be amorphous, as can be seen in the blue-framed FFT patterns in Fig. 3c–f. Interestingly, no clear lattice fringe was observed in the CF region, indicating the CF was weakly crystallized or amorphous, which was quite different from the learned knowledge that the CF was with crystalline metallic phase^16^,^17^,^18^. From the refined FFT patterns (shown in the upper inserts of Fig. 3c), the CF edges presented amorphous phases, whereas crystalline phase was detected in the CF center. The measured fringe space (0.2 nm) matched with the d-space of <111> plane of face-centered cubic copper. These findings suggest a possible scenario, i. e. the incorporated Cu element is merged with the amorphous HfO_2_. If the CF is composed by pure Cu, the original HfO_2_ material in the CF region should be pushed aside. In such a case, serious structure deformation should take place, however, from the TEM image, no serious deformation around the CF was detected. This result suggests the incorporated Cu element occupy some places in HfO_2_ lattice. Similar results were also found in Ag/SiO_2_ system^17^,^18^, where the Ag cluster was detected in SiO_2_ material with Ag atoms accumulated in the void position. The movement of Ag cluster from one site to another contributed to the formation of conductive filament. One point should be mentioned that no crystalline Ag was found in the space between the clusters, suggesting the Ag actually be merged with the SiO_2_ lattice, consistent with the result in this work. According to the formation energy of a Cu atom in the HfO_2_ lattice, the Cu atom is more likely to occupy the interstitial sites of the HfO_2_ matrix, rather than the substitutional sites^35^,^36^. The growth of filament relies on the transportation of Cu element to the next adjacent interstitial site.

## Disscussion

**Figure 4:**
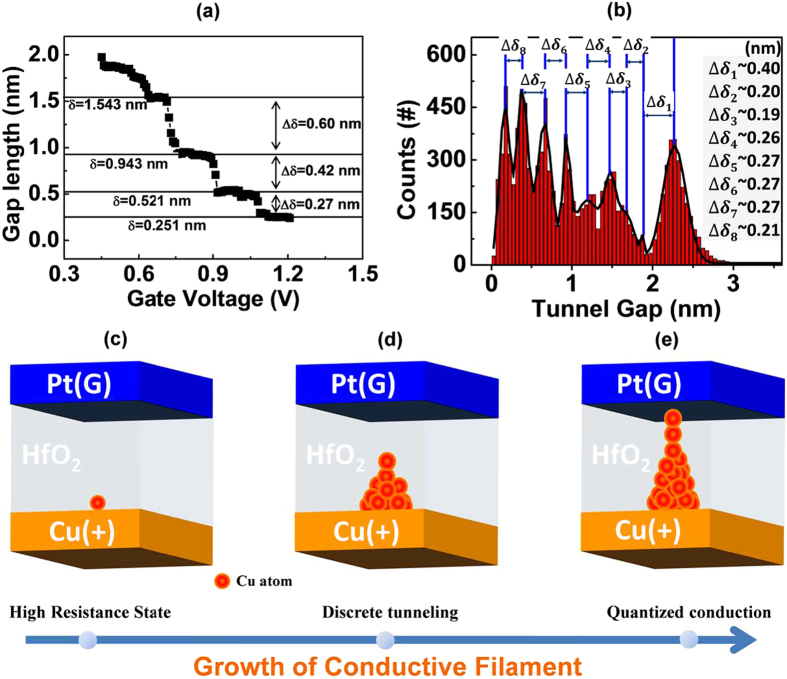
Description of the discrete resistance change using direct tunneling. (**a**) The gap length vs. the V_G_ curve fitted by the low voltage tunnel equation. The barrier height is 2.0 eV and the size of the filament tip is 2.5 nm in calculation. (**b**) Histogram of the tunnel gap lengths obtained from the resistance data in Fig. 2(b). (**c**–**e**) Schematic diagrams of filament growth.

Based on the tunneling conduction during filament growth, the filament length can be easily correlated with the measured resistance by a simple low voltage direct tunnel equation (the programming voltage was generally less than 0.5 V, as shown in Figure S6). This tunneling system had three components (the filament tip, tunnel gap and counter electrode) and three key parameters (the tip size, barrier height and barrier width). The barrier height for electron transmission was assigned to 2.0 eV, which was estimated from the I-V curve of a fresh device with a known barrier width of 3 nm (Figure S7). Although the real situation is more complex than the fresh state, the value of the barrier height was reasonable and comparable with the reported results^37^,^38^,^39^. Another parameter related to tunnel resistance is the area of the filament tip. Most studies have assumed the tip size to be 1 to 4 nm^27^,^40^. After establishing the two critical parameters, the cell resistance can then be regenerated by calculating the tunnel resistance with the variable gap length or barrier width. The modeling data are shown in Figure S8. The relationship between R and V_G_, as shown in Fig. 2a, can be converted to the gap length vs. V_G_, as shown in Fig. 4a. Discrete tunneling corresponds to discrete increase of the filament. Interestingly, the increment of filament was found with a feature of basic unit (0.2 nm and 0.27 nm from the above modeling parameters). Moreover, this unit length was nearly independent of the size of filament tip, as shown in Figure S9. Figure 4b shows the histogram of the tunnel gap lengths corresponding to the data in Fig. 2b. The difference between two neighboring peaks of the gap length was either 0.2 nm or 0.27 nm.

According to the HRTEM results, the Cu element is more likely merged with the lattice of HfO_2_. The distance between two adjacent interstitial sites in the HfO_2_ lattice is about 0.25 nm, which is very close to the observed unit length of CF growth. Based on the above findings, we can draw a clear picture of filament growth. Under high electric field, Cu atoms in the active electrode will first be oxidized to Cu ions and injected into the HfO_2_ layer. The Cu ions then occupy the interstitial sites of the HfO_2_ lattice. However, due to low cation mobility, the Cu ions can only travel a short distance before they are reduced by the oncoming electrons. The subsequently injected Cu ions then move to the next hopping site and are reduced again. The growth of filament relies on the subsequently injected Cu ions, which move to the next adjacent interstitial site and are reduced again. Under the repetitive ion transportation and localized reduction, the filament grows from the active electrode toward the inert electrode. Figure 4c–e provide schematics of filament growth.

In summary, the kinetics of filament growth in a Cu/HfO_2_/Pt device was investigated. Detailed information on the CF growth beneath the fast resistive switching was revealed using a refined real-time current regulation scheme in a 1T1R structure. Discrete tunneling conduction and quantized conduction were observed during programming. Based on direct tunneling conduction, the filament was found to increase with a length of unit feature, matching with the hopping conduction mechanism of Cu ions between the interstitial sites of HfO_2_ lattice. The physical nature of the formed CF was characterized by HRTEM. Copper rich conical CF was directly identified. The incorporated Cu element was found merged with the amorphous HfO_2_, in good accordance with the observation of discrete tunneling behavior. This work provides a comprehensive understanding of the resistance change of oxide electrolyte based electrochemical memristive elements.

## Methods

### Fabrication of 1T1R memristive cells

The N type transistor was fabricated by a standard 0.13 μm logic process. Memristive devices with Cu/HfO_x_/Pt structures were integrated on it. A Cu plug was chemical-mechanical polished, then used as the bottom electrode. The HfO_x_ electrolyte layer and Pt top electrode were grown by successive ion beam sputtering and electron-beam evaporation at room temperature, with thicknesses of 4 nm and 70 nm, respectively. The cell size was about 300 nm × 400 nm, defined by the bottom Cu plug. The channel width/length of the selector transistor was 10 μm/1 μm.

### Characterization

The electro-characterization of the 1T1R memristive device was performed with a Keithely 4200 SCS semiconductor parameter analyzer. The current during the SET transition was limited by properly biasing the gate voltage on the selector transistor. The voltage drop on the memristive cell was obtained from the output voltage of the bottom electrode. The TEM specimen was prepared by focused ion beam cutting and milling. The material compositions were analyzed by energy-dispersive spectroscopy.

## Additional Information

**How to cite this article**: Lv, H. *et al.* Atomic View of Filament Growth in Electrochemical Memristive Elements. *Sci. Rep.* **5**, 13311; doi: 10.1038/srep13311 (2015).

## Supplementary Material

Supplementary Information
