# Supplementary material for: Atomic View of Filament Growth in Electrochemical Memristive Elements
Source: Sci Rep. 2015 Aug 21;5:13311. doi: 10.1038/srep13311 (PMC4543950; doi:10.1038/srep13311)
Supplement: Supplementary Information [file srep13311-s1.pdf]

Supplementary Information

Atomic View of Filament Growth in Electrochemical Memristive Elements

Hangbing Lv[1, 2], Xiaoxin Xu[1, 2], Pengxiao Sun[1, 2], Hongtao Liu[1, 2], Qing Luo[1, 2], Qi Liu[1, 2], Writam Banerjee[2], Haitao Sun[1, 2], Shibing Long[1, 2], Ling Li[1, 2] & Ming Liu*[1, 2]

[1]Key Laboratory of Microelectronics Devices and Integrated Technology, Institute of Microelectronics, Chinese Academy of Sciences, Beijing, 100029, China;

[2]Lab of Nanofabrication and Novel Devices Integration Technology, Institute of Microelectronics, Chinese Academy of Sciences, Beijing, 100029, China

Correspondence and requests for materials should be addressed to M.L. (email: liuming@ime.ac.cn)

Table of Contents:

1. **Device structure and equivalent circuit of 1T1R cell**
2. **Multi-level storage achieved by the varied gate voltage**
3. **Temperature dependence of the cell resistance for V_G<1.1V**
4. **Estimation of the serial resistance on the quantized conduction**
5. **EDS spectrums of CF region and non-CF region**
6. **The voltage drop on cell during programming**
7. **Estimation of barrier height for electron transmission**
8. **The simulation result of tunnel resistance versus gap length**
9. **Calculation of the filament length with different tip sizes**

S1. Device structure and equivalent circuit of 1T1R cell.

(a)

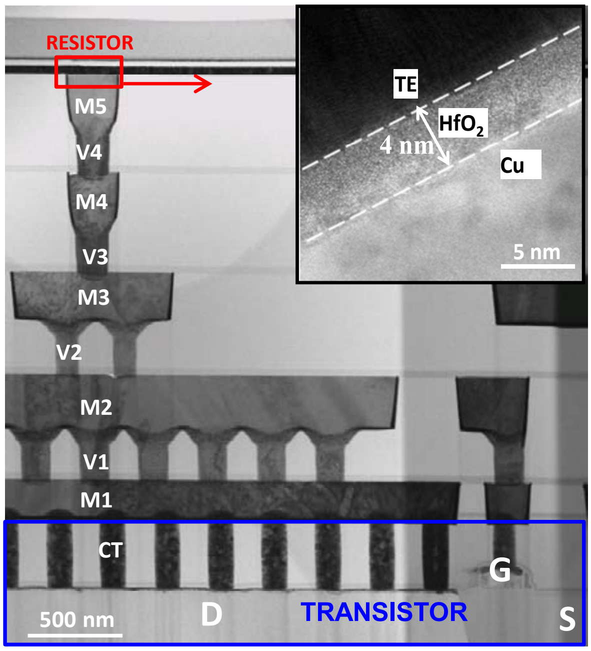

(b)

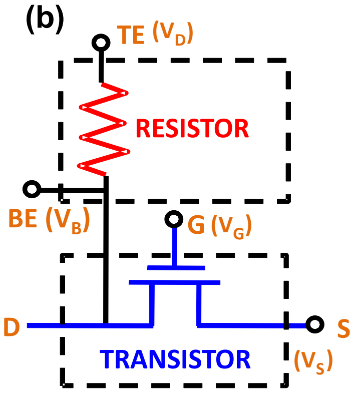

(c)

	HRS to LRS	LRS to HRS
V_D	GND	+
V_G	+	+
V_S	+	GND

Figure S1. (a) The cross-section TEM image of 1T1R structure. The memristive element with Cu/HfO2/Pt structure is fabricated on the top of the transistor. The Cu plug, which connects the drain of transistor, serves as bottom electrode after CMP. The insert is magnification of Cu/HfO_2/Pt structure. The HfO_2 layer is about 4 nm. (b) Equivalent circuit of 1T1R cell. (c) Operation conditions. Positive voltage bias on Cu electrode is needed to perform the forming and SET operation with the Pt electrode grounded. For RESET operation, the voltage polarity should be opposite. In the case of 1T1R structure here, the V_S and V_D should be positively biased for forming/SET and RESET, respectively.

S2. Multi-level storage achieved by the varied gate voltage.

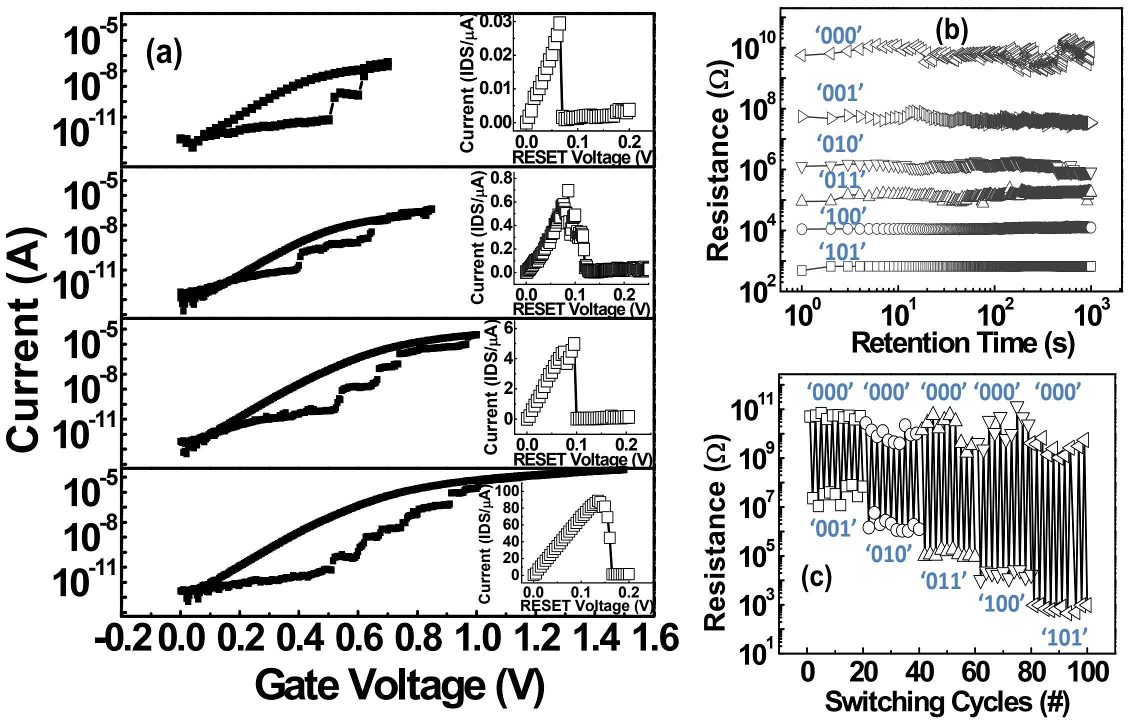

Figure S2. (a) The curves of I_{ds} verse V_G. The cell resistance can be precisely controlled by different V_G. The inserts are the I-V curves of corresponding RESET operation. Ultra low switching current of nA level has been realized. (b) Retention characteristics of multi resistance states. (c) Repetitive switching cycles between different resistance states.

S3. Temperature dependence of the cell resistance for V_G<1.1V.

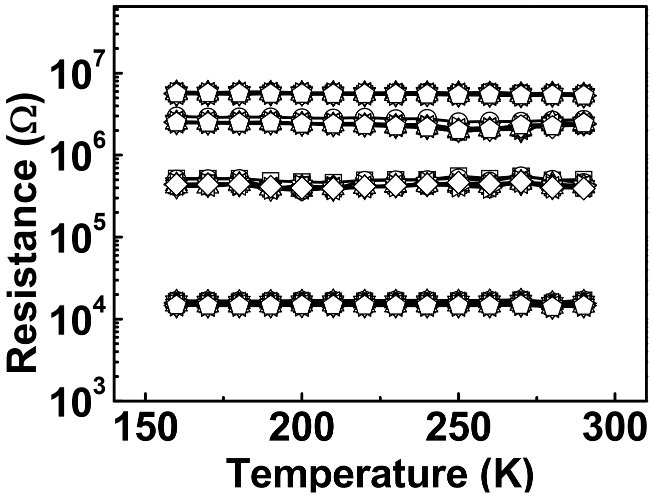

Figure S3. The cell resistance programmed achieved by $V_G<1.1$V. The independence of resistance on temperature was observed, indicating tunneling was the dominant conduction in these resistance states.

S4. Estimation of the serial resistance on the quantized conduction.

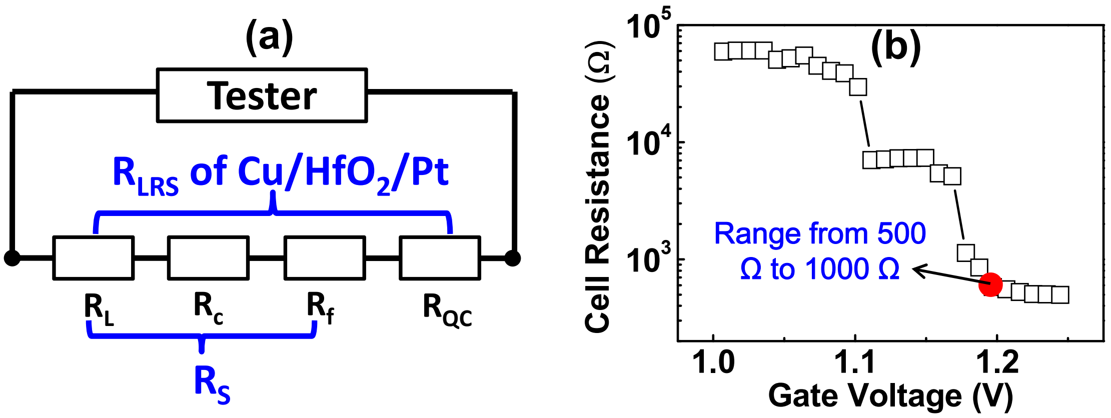

Figure S4. (a) Schematic of components of serial resistance (R_S). R_L represents the line resistance in testing environment. R_f is the resistance of filament from Cu side to its tip. R_c is the contact resistance between filament and Cu electrode. (b) The cell resistance for $1V<V_G<1.25V$. The red dot represents the formation of complete filament. The resistance at this point can be approximately

considered as a sum of R_L, R_c and R_f. The typical value of R_S ranges from 500 Ω to 1000 Ω.

S5. EDS spectrums of CF region and non-CF region.

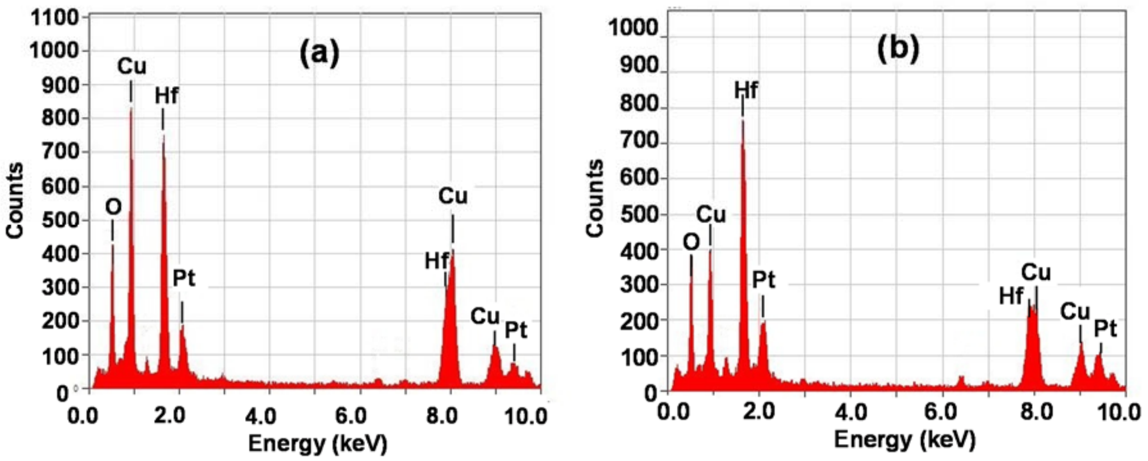

Figure S5. (a) CF region. (b) Non-CF region. The feature peaks of Cu signal in CF region is much higher than that in non-CF regions.

S6.The voltage drop on cell during programming.

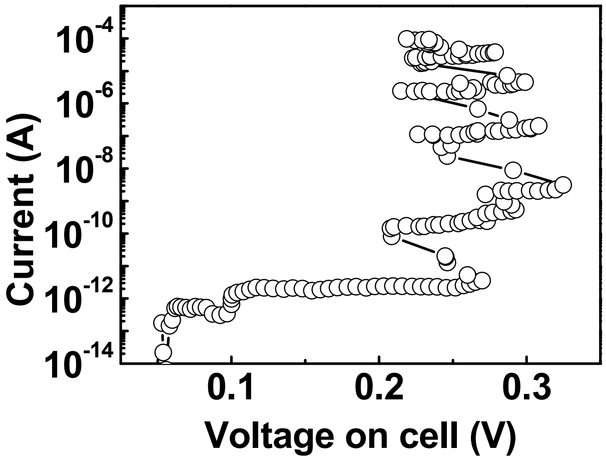

Figure S6. The V_C can be directly detected by outputting the voltage of the bottom electrode.

S7. Estimation of barrier height for electron transmission.

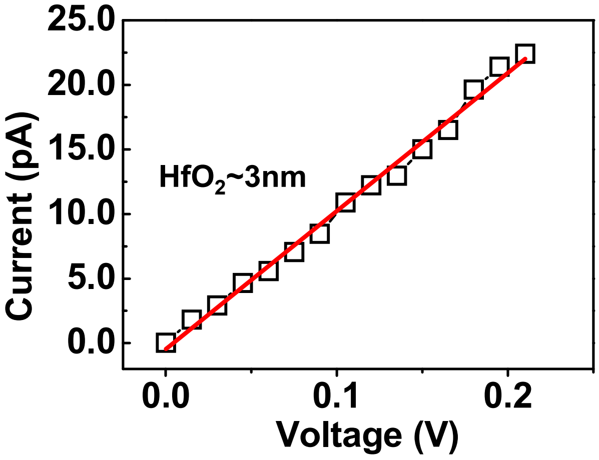

Figure S7. The I-V curve of fresh device with 3 nm HfO_2. The tunneling current from the filament tip to counter electrode can be expressed by: $I = A\frac{\sqrt{\bar{\phi}}}{\delta}\exp(-\frac{4\pi\delta}{h}\sqrt{2m_{eff}\bar{\phi}})V$, where A is the area of tip, $\bar{\phi}$ is the barrier height, δ is the barrier width (gap length), m_{eff} is the effective mass of electron and h is the Planck constant. For the fresh cell, A is around 300 nm × 400 nm, δ =3 nm, and m_{eff} =0.18m₀. (ref. IEEE TED 2011, 58, 2878.) The barrier height $\bar{\phi}$ (2.0 eV) can be obtained by fitting the I-V curve.

S8. The simulation result of tunnel resistance versus gap length.

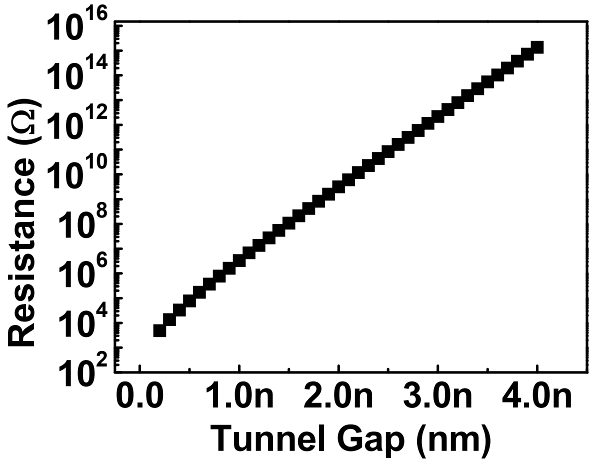

Figure S8. The resistance of tunnel gap, which can be calculated on the base of direct tunneling

equation, with CF tip of 2.5 nm, $\bar{\phi}$=2.0 eV and m_{eff} =0.18m$_0$.

S9. Calculation of the filament length with different tip sizes.

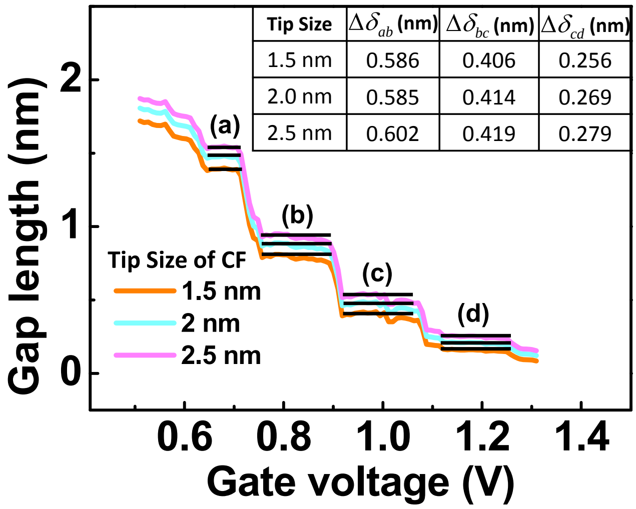

Figure S9. The relation of filament length with the gate voltage on the case of filament with different tip sizes. The increment of filament length was nearly independent of the tip size.
